# Acupuncture and related therapies for carpal tunnel syndrome: A protocol for systematic review and Bayesian network meta-analysis

**DOI:** 10.1097/MD.0000000000028294

**Published:** 2021-12-17

**Authors:** Zhiyuan Bian, Jie Yu, Mingqi Tu, Binjun Liao, Jingmei Huang, Yongliang Jiang, Jianqiao Fang

**Affiliations:** a Department of Neurobiology and Acupuncture Research, the Third Clinical Medical College, Zhejiang Chinese Medical University, Key Laboratory of Acupuncture and Neurology of Zhejiang Province, Hangzhou, China; b Department of Acupuncture and Massage, Affiliated Hangzhou First People's Hospital, Zhejiang University School of Medicine, Hangzhou, China.

**Keywords:** acupuncture, carpal tunnel syndrome, network meta-analysis

## Abstract

**Background:**

Carpal tunnel syndrome (CTS) is the most common peripheral nerve compression syndrome of the upper limb. Plenty of studies showed the effects of acupuncture therapy on relieving pain and improving functional status for CTS patients. Diverse types of acupuncture therapies have been used in the treatment for CTS, but their relative treatment effects are poorly understood. This study will evaluate the effects of different acupuncture and related therapies for CTS by conducting a systematic review and Bayesian network meta-analysis (NMA).

**Methods:**

We will search randomized controlled trials (RCTs) of acupuncture and related therapies for CTS in MEDLINE (via PubMed), EMBASE, Web of Science, Cochrane Library, Chinese Biomedical Database, China National Knowledge Infrastructure, VIP Database, Wanfang Database, WHO International Clinical Trials Registry Platform, ClinicalTrials.gov, Chinese Clinical Trial Register, and OpenGrey from inception to November 2021. Then, we will select eligible studies, extract data, and conduct risk of bias assessment using the Cochrane tool. Pairwise meta-analysis and Bayesian NMA will be performed in Stata 15.1 software and Aggregate Data Drug Information System 1.16.8 software. We will assess the quality of the evidence using the Confidence in Network Meta-Analysis application.

**Results:**

In this study, the treatment effects and safety of different acupuncture and related therapies for CTS will be evaluated.

**Conclusion:**

This study will provide evidence for choosing the optimal acupuncture and related therapies in the treatment for CTS.

## 1 Introduction

Carpal tunnel syndrome (CTS) is the most common nerve entrapment syndrome of the upper limb, which is caused by the mechanical compression and ischemia-mediated impairment to the median nerve within the carpal tunnel.^[1,2]^ The reported prevalence of CTS varies according to different diagnostic criteria (e.g., clinical or electrophysiological criteria). In general population, the CTS prevalence is approximately 5%, and studies based on working population in US and China showed a higher prevalence at 7.8% and 9.6%, respectively.^[3–5]^ Patients with CTS typically present pain or paresthesia in distribution of median nerve distal to the wrist, and symptoms may also happen in forearm, upper arm, and shoulder.^[6]^ These symptoms appear in a relapsing-remitting pattern or gradually worsen, triggered by daily life activities such as lifting and computer use. Intermittent symptoms may progress to continuous pain, weakness, and thenar atrophy, which could greatly affect patients’ life.^[7]^ Diverse types of nonoperative treatments and surgical options are available in the management of CTS. Splinting and local corticosteroid injection are considered as practical treatments in the primary care setting, whereas studies showed the effectiveness in long term follow-up remains unclear.^[3,8,9]^ Surgical decompression is a well-established treatment for CTS, with reported longer lasting effect comparing to corticosteroid injection.^[6]^ However, surgical treatment involves potentially more complications, including scar tenderness, pillar pain, and transient neuropraxia.^[10]^

Acupuncture is an important part of traditional Chinese medicine (TCM), which has been practiced in China for thousands of years. As acupuncture therapy gains increasing popularity worldwide, it is considered to be an effective and safe complementary treatment for plenty of diseases, especially for pain conditions.^[11,12]^ In China, diverse acupuncture therapies based on TCM theory have been used in the treatment for CTS, such as manual acupuncture, moxibustion, warm acupuncture, etc. In addition, acupotomy, a modern type of acupuncture using a blade-needle with surgical scalpel at its tip, has also been widely used for CTS.^[13]^ The existing clinical trials and reviews have shown the effect of acupuncture therapies on symptom relief for CTS patients, but the relative treatment effects of different acupuncture therapies are unclear.^[14–16]^ In this study, we aim to compare the efficacy and safety of different acupuncture and related therapies for CTS using systematic review and network meta-analysis (NMA).

## 2 Methods

### 2.1 Study registration

This protocol of systematic review and NMA has been registered in the INPLASY website (registration number: INPLASY2021110094). This study protocol will be presented under the guidance of the Preferred Reporting Items for Systematic Review and Meta-Analysis Protocols (PRISMA-P) statement.^[17]^

### 2.2 Inclusion criteria for study selection

#### 2.2.1 Types of studies

All randomized controlled trials (RCTs) with a parallel-group design on acupuncture and related therapies for CTS. This study will not include quasi-RCTs, cross-over trials, cluster RCTs, or any type of non-RCTs. The reporting language is limited to English and Chinese.

#### 2.2.2 Types of participants

Patients who were diagnosed with CTS. No limitation on age, sex, or race of patients will be required. However, this study will not include CTS patients who have been treated by surgical decompression.

#### 2.2.3 Type of interventions

In intervention group, patients will be treated with acupuncture and related therapies (manual acupuncture, moxibustion, electroacupuncture, warm needling, acupotomy, etc), combinations of different acupuncture therapies, or combinations of acupuncture therapies with other conservative treatments. There is no restriction on manual acupuncture techniques, moxibustion methods, acupoint selection, needle materials, treatment duration, and session. However, this study will exclude acupoint injection treatment because it may involve with the use of drugs that are similar to other injection therapy. In comparison group, patients will be treated with other conservative treatments (splinting, corticosteroid injections, laser therapy, etc), placebo, sham acupuncture, or different acupuncture therapies (combinations) from the intervention group. This study will not include trials involving with herbal medicine.

#### 2.2.4 Types of outcome measures

The primary outcomes will be CTS related symptom and function status measured by the Boston carpal tunnel syndrome questionnaire (BCTQ), which consists of the symptom severity scale (SSS) and function severity scale (FSS) or other rating scales, and CTS related pain intensity measured by Visual Analogue Scale (VAS) or other scales. The secondary outcomes will be response rate, electrophysiological status of median nerve, and adverse events happened during the treatment period.

### 2.3 Search methods for identification of studies

We will search relevant studies in the following databases from inception to November 2021: MEDLINE (via PubMed), EMBASE, Web of Science, Cochrane Library, Chinese Biomedical Database, China National Knowledge Infrastructure, VIP Database, Wanfang Database, WHO International Clinical Trials Registry Platform, ClinicalTrials.gov, Chinese Clinical Trial Register, and OpenGrey. The searching languages will be limited to either English or Chinese. Search terms will include medical subject headings terms (MeSH) and free-text terms, which can be categorized into clinical condition, interventions, and study design. Reference lists of included studies will also be reviewed to identify potential eligible studies. The search strategy for PubMed is presented in Table 1.

**Table 1:** Search strategy in PubMed.

Order	Search items
#1	MeSH Terms: “Carpal Tunnel Syndrome” OR “Median Nerve” OR “Median Neuropathy”
#2	Title/Abstract: “carpal tunnel syndrome” OR “median nerve” OR “median neuropathy” OR “median neuritis” OR “CTS”
#3	#1 OR #2
#4	MeSH Terms: “Acupuncture Therapy” OR “Acupuncture” OR “Moxibustion” OR “Electroacupuncture” OR “Cupping Therapy” OR “Bloodletting”
#5	Title/Abstract: “acupuncture” OR “electroacupuncture” OR “moxibustion” OR “moxa” OR “acupotomy” OR “acupotome” OR “acupressure” OR “cupping” OR “bloodletting” OR “blood-letting” OR “pyonex” OR “pricking blood” OR “needle” OR “needles” OR “needling” OR “acupoint” OR “acupoints” OR “meridian” OR “meridians”
#6	#4 OR #5
#7	Publication Type: “Randomized Controlled Trial”
#8	MeSH Terms: “Randomized Controlled Trials as Topic”
#9	Title/Abstract: “randomized” OR “randomly” OR “RCT” OR “trial”
#10	#7 OR #8 OR #9
#11	#3 AND #6 AND #10

### 2.4 Data collection and analysis

#### 2.4.1 Studies selection

**Figure 1:**
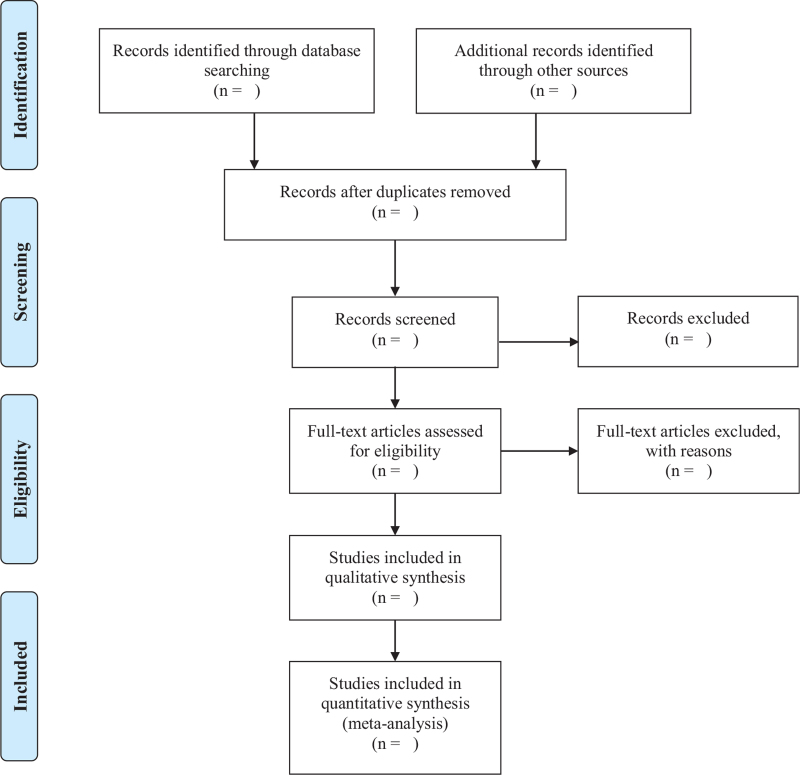
PRISMA flow diagram. PRISMA = Preferred Reporting Items for Systematic review and Meta-Analysis.

Two researchers will screen the titles and abstracts of retrieved references independently after removing duplicate literature using Noteexpress 3.2.0 software. Then, full texts of potential eligible studies will be downloaded for further evaluation according to the inclusion criteria. Reviewers will contact the study authors if there is incomplete information. Discrepancies between these 2 researchers will be solved by discussion with a third researcher. The reason of exclusion for each excluded study will be recorded. The study selection process will be summarized in a Preferred Reporting Items for Systematic Reviews and Meta-Analyses (PRISMA) flowchart (Fig. 1).^[18]^

#### 2.4.2 Data extraction

Two reviewers will independently collect information from included studies using a pre-designed form. Following contents will be extracted: publication year, authors, characteristics of participants, sample size, details of interventions and comparisons including types of acupuncture therapy, acupoints selection, drug names, dosage, route of administration, treatment frequency and duration, number of sessions, and outcomes. Cross-check will be done and any disagreement will be solved by discussion with a third reviewer.

#### 2.4.3 Dealing with missing data

If there is missing data of included trial, the corresponding author of the original study will be e-mailed to obtain the relevant data. In the case of no reply, absent data relating to the quantitative synthesis will be calculated using specific formula based on available data.^[19]^ If data cannot be obtained after these steps, the study with incomplete data will be excluded for data synthesis.

#### 2.4.4 Assessment of risk of bias

The methodological quality of each included trial will be assessed using the Cochrane Collaboration Risk of Bias Tool, which comprises following domains: random sequence generation, allocation concealment, blinding of participants and personnel, blinding of outcome assessment, incomplete outcome data, selective reporting, and other sources of bias.^[20]^ Two reviewers will independently rate each of these domains as high, low, or unclear risk of bias, and disagreement will be solved by consulting to a third reviewer. Finally, an overall judgment of risk of bias for each study will be generated.

### 2.5 Data synthesis

#### 2.5.1 Pairwise meta-analysis

For a certain outcome, when at least 2 studies comparing the same pair of interventions exist, the standard pairwise meta-analysis will be conducted. The fixed-effects model using the Mantel–Haenszel method or random-effects model using the DerSimonian-Laired method will be fitted in Stata 15.1 software. We will use *I*^2^ statistic to measure the statistical heterogeneity. When *I*^2^ <50%, a fixed-effects model will be used, otherwise, a random-effects model will be chosen. For continuous outcome, mean difference (MD) or standardized mean difference (SMD) on the change score (pre-post difference) with 95% confidence interval (95% CI) will be used. And for dichotomous outcome, odds ratio (OR) with 95% CI will be used.

#### 2.5.2 Network meta-analysis

Network plot of all included interventions for each outcome will be generated in Stata 15.1 software. We will conduct a Bayesian NMA using the Aggregate Data Drug Information System 1.16.8 software with Markov Chain Monte Carlo (MCMC) simulation. Four MCMC chains will be set with 20,000 tuning iterations and 50,000 simulation iterations. The convergence of the model will be assessed by the Brooks-Gelman-Rubin method. When potential scale reduced factor (PSRF) is close to 1, the convergence is considered achieved. The relative effect of interventions will be presented with 95% credible interval (CrI). Node-splitting method will be used to evaluate the inconsistency between direct evidence and indirect evidence.^[21]^ For each outcome, a rank probability of all included interventions will be generated.

#### 2.5.3 Subgroup and sensitivity analyses

If significant heterogeneity is detected, the potential source of heterogeneity will be explored by conducting meta-regression or subgroup analysis. If there is severe heterogeneity and reviewers cannot identify the source of heterogeneity, a narrative summary of finding will be presented. Sensitivity analysis will be performed by removing studies rated as high risk of bias to assess the stability of the result.

#### 2.5.4 Assessment of publication bias

The comparison-adjusted funnel plot will be used to assess potential publication bias when sufficient studies are included in the quantitative synthesis.^[22]^

### 2.6 Assessment of the evidence quality

The quality of the result from NMA will be assessed using the Confidence in Network Meta-Analysis (CINeMA) application.^[23]^ Following domains will be evaluated by 2 independent researchers: within-study bias, across-study bias, indirectness, imprecision, heterogeneity, and incoherence. The evidence quality will be determined as high, moderate, low, or very low. Any disagreement will be solved by discussion with a third reviewer.

## 3 Discussion

CTS is a common hand disorder which largely influences people's daily activities. Although both surgical and nonsurgical treatments are available, clinical issues such as surgical complications, limited effect of repetitive use of steroid injections and recurrence of symptoms are still disturbing.^[3]^ Plenty of complementary therapies have been proposed in the treatment for CTS.^[6,24]^ Acupuncture, an increasingly used therapy in pain management with little risk of adverse effects, has also been practiced for treating CTS.^[25]^ Preclinical researches have shown acupuncture can block different types of pain through peripheral and central mechanisms, and it can induce anti-inflammatory responses.^[26,27]^ In China, many different types of acupuncture for treating CTS have been reported. The majority of current clinical trials of acupuncture for CTS focus on comparing acupuncture therapies with conventional conservative treatments, whereas the relative treatment effects of different acupuncture therapies are less investigated.

NMA can compare the effects of multiple interventions simultaneously, by combining direct and indirect evidence,^[28]^ and within a Bayesian framework, the ranking probability of the treatment effects can be obtained. We plan to conduct a systematic review and Bayesian NMA to compare different acupuncture and related therapies for treating CTS. However, this study has some limitations: we will only include studies reported in English and Chinese, which may lead to language bias; this NMA will focus on comparing different types of acupuncture therapies, without the consideration on other detail of the treatment, such as selection of acupoints and manual techniques. The result of this study will provide reliable and practical suggestions for acupuncture practitioners in clinical decision-making.

## Author contributions

**Conceptualization:** Zhiyuan Bian, Jie Yu.

**Data curation:** Mingqi Tu, Binjun Liao.

**Formal analysis:** Binjun Liao, Jingmei Huang.

**Investigation:** Zhiyuan Bian, Jingmei Huang.

**Methodology:** Zhiyuan Bian, Jie Yu.

**Software:** Mingqi Tu, Binjun Liao.

**Supervision:** Yongliang Jiang, Jianqiao Fang.

**Writing – original draft:** Zhiyuan Bian, Mingqi Tu.

**Writing – review & editing:** Yongliang Jiang, Jianqiao Fang.
